# Protecting intellectual property associated with Canadian academic clinical trials - approaches and impact

**DOI:** 10.1186/1745-6215-13-243

**Published:** 2012-12-27

**Authors:** Sue Ross, Laura Magee, Mark Walker, Stephen Wood

**Affiliations:** 1 Department of Obstetrics and Gynaecology, University of Calgary, 1441 29th Street NW, Calgary, T2N 4J8, Canada; 2 Department of Community Health Sciences, University of Calgary, 3280 Hospital Drive NW, Calgary, T2N 4Z6, Canada; 3 Department of Family Medicine, University of Calgary, 3330 Hospital Drive NW, Calgary, T2N 4N1, Canada; 4 Department of Surgery, University of Calgary, 1403 29th Street NW, Calgary, T2N 2T9, Canada; 5 Department of Obstetrics and Gynecology, University of Alberta, 10240 Kingway Avenue, Edmonton, T5H 3V9, Canada; 6 Department of Medicine, University of British Columbia, 4500 Oak Street, Vancouver, V6H 3N1, Canada; 7 Ottawa Hospital Research Institute, University of Ottawa, 1053 Carling Avenue, Ottawa, K1Y 4E9, Canada

**Keywords:** Clinical trials as topic, Intellectual property, Trial protocol

## Abstract

Intellectual property is associated with the creative work needed to design clinical trials. Two approaches have developed to protect the intellectual property associated with multicentre trial protocols prior to site initiation. The ‘open access’ approach involves publishing the protocol, permitting easy access to the complete protocol. The main advantages of the open access approach are that the protocol is freely available to all stakeholders, permitting them to discuss the protocol widely with colleagues, assess the quality and rigour of the protocol, determine the feasibility of conducting the trial at their centre, and after trial completion, to evaluate the reported findings based on a full understanding of the protocol. The main potential disadvantage of this approach is the potential for plagiarism; however if that occurred, it should be easy to identify because of the open access to the original trial protocol, as well as ensure that appropriate sanctions are used to deal with plagiarism. The ‘restricted access’ approach involves the use of non-disclosure agreements, legal documents that must be signed between the trial lead centre and collaborative sites. Potential sites must guarantee they will not disclose any details of the study before they are permitted to access the protocol. The main advantages of the restricted access approach are for the lead institution and nominated principal investigator, who protect their intellectual property associated with the trial. The main disadvantages are that ownership of the protocol and intellectual property is assigned to the lead institution; defining who ‘needs to know’ about the study protocol is difficult; and the use of non-disclosure agreements involves review by lawyers and institutional representatives at each site before access is permitted to the protocol, significantly delaying study implementation and adding substantial indirect costs to research institutes. This extra step may discourage sites from joining a trial. It is possible that the restricted access approach may contribute to the failure of well-designed trials without any significant benefit in protecting intellectual property. Funding agencies should formalize rules around open versus restricted access to the study protocol just as they have around open access to results.

## Background

### Why intellectual property is becoming increasingly important

Academic institutions and their affiliated investigators are encountering a rapidly changing research environment, in which larger numbers of researchers and universities are competing for increasingly limited government [1], research trust and industry funding. In this competitive environment, securing research funding for investigator-initiated clinical trials depends not only on the quality of grant applications and individual researchers, but also on the reputation of the academic institutions themselves [2]. The reputation and wealth-generating potential of institutions are influenced by the creative output generated by faculty members, as well as the ownership and management of the intellectual property (IP) associated with that creative output [2-5].

This paper discusses the intellectual property associated with the design of investigator-initiated clinical trial protocols as a specific example of creative work, and a trend appearing in which institutions attempt to prevent expropriation of that intellectual property.

### Protecting intellectual property

Institutions take a variety of approaches to the management and protection of intellectual property. In the case of investigator-initiated multicentre academic trials, investigators and their institutions have until recently been complacent about protecting their intellectual property, at least partly because it is difficult to identify the intellectual property associated with such trials. With the advent of open access journals and easy access to the internet and trial websites and registries, a commonly used strategy is to publish the trial protocol. This ‘open access’ approach is easily recognised by most producers and users of research evidence, because their main access to research reports is through publications available in journals and from online sources.

Another recently noted strategy in Canada is the use of non-disclosure agreements (NDAs) to protect the details of study protocols. NDAs are legal documents developed by the lead academic institution (the ‘sponsor’ of the research). NDAs must be signed by the site investigator and an institutional representative of any potential colla-borating site before they are given access to the research protocol, a ‘restricted access’ approach. The goal of NDAs is to limit access to ‘proprietary information’, in this case the details of the study protocol, to those individuals (‘employees and agents’) who need to have those details of the trial before their site decides whether to join the trial. Should the site investigator or other individual breach the agreement, ‘remedies at law’ may be pursued by the lead academic institution.

NDAs originated in industry trials. In that instance, the manufacturer wishes to protect proprietary information contained in a protocol, for example details of a new drug product, that they do not wish to become widely available. This is not the case with investigator-initiated trials, which tend to evaluate treatments or treatment policies that do not require such protection, and a rigorous protocol is generally composed of elements that are already validated and published (and as such, are in the public domain). For example, a rigorous study design will use valid and reliable methods for measuring the outcome of treatments or interventions. Similarly, trial designs will use previously published methods for subject randomisation, statistical analysis and other aspects of the study design. The intellectual property associated with an investigator-initiated trial protocol is therefore related to the complete package of trial interventions and procedures.

### What this paper is about

This commentary explores the contrasting approaches to protection of intellectual property (by permitting open versus restricted access to protocols) taking as examples two multicentre investigator-initiated trials currently being conducted in Canada. The two trials are ongoing international perinatal trials, both funded by the Canadian Institutes of Health Research and registered with the International Standard Randomised Controlled Trial Number Register [6]. Both trials are led by sophisticated trials units incorporating the highest standard of data management and monitoring, and involve large networks of committed investigators. Our commentary is based on documents that are publicly available.

### Examples of two trials

#### CHIPS (control of hypertension in pregnancy study)

CHIPS [7] is led by the University of British Columbia Maternal Fetal Medicine Division of the Department of Obstetrics and Gynaecology and the Sunnybrook Research Institute Centre for Mother, Infant and Child Research (CMICR). The trial will recruit 1,028 pregnant women in 19 countries. Women with hypertension are randomised to ‘less tight’ or ‘tight’ control of hypertension. The primary outcome is a composite of pregnancy loss plus neonatal intensive care.

In this trial, the investigators safeguarded intellectual property through open access to the study protocol. The full protocol was made available online [8] and a brief summary could be found at *The Lancet* website [9].

#### FACT (folic acid clinical trial)

FACT [10], led by the Ottawa Hospital Research Institute is recruiting 3,656 pregnant women in seven countries. Pregnant women are randomised to receive either 4 mg folic acid or placebo daily. The study primary outcome is development of pre-eclampsia.

When the trial first started, intellectual property was safeguarded by requiring potential local investigators and institutions to sign NDAs before the full protocol was provided for their consideration. Due to the delays in implementation, frustration of collaborators and excess use of resources in this process both in the lead trial center and for other research institutes, this policy was later abandoned.

#### Main advantages and disadvantages of each approach

The advantages and disadvantages for each approach are summarised for each stakeholder in Table 1. The main stakeholders included are the lead investigators and their institutions, funding bodies, site collaborators and their institutions, end users of research, actual and potential participants, and society more generally. (An NDA refers to protection of the protocol before a centre joins a trial, but this analysis assumes ongoing protection of the protocol during the conduct of the trial, once a sub-site agreement has been signed).

**Table 1:** Stakeholder perspectives on open versus restricted access to trial protocols

**Stakeholder**	**Open access** (**online protocol**)		**Restricted access** (**NDA**)*	
	**Advantages**	**Disadvantages**	**Advantages**	**Disadvantages**
**Lead investigators**	· Authorship acknowledged.	· Investigators do not have control over who has access to the protocol.	· Ownership established by the lead centre.	· Cost and time involved in establishing and defending NDAs.
	· Plagiarism can be identified.			
			· Potential for plagiarism reduced.	
	· Investigators must adhere to protocol, or explain reasons why not.	· Protocol could be adapted or plagiarized by other investigators.		
		· Need to report all amendments to protocol.		
	· High degree of rigour associated with adhering to protocol and reporting.			
				· The lead investigator is the only one given credit for the protocol; other investigators do not receive adequate recognition of the effort that they have expended in developing the protocol.
				· Co-investigators in other centres will need to sign NDAs before being able to establish the trial in their centre.
			· Investigators might be less accountable for changes to protocol	
**Investigators**’ **institutions** (**universities**, **health care organisations**)	· Affiliation acknowledged.	· Institution does not have control over who has access to the protocol.	· Ownership by institution established.	· Cost involved in establishing and defending NDAs.
**Peer**-**reviewed funding bodies**	· Public knowledge of funded study (that is, use of funding).	· Protocol could be adapted or plagiarized by other investigators.	· Confidence that protocol will not be plagiarized.	· Public cannot access study protocol.
				· Less certainty that study will be conducted as funded.
	· Greater accountability of investigators to adhere to funded protocol.			
	· Greater certainty that study will be conducted as funded.			
**Industry funding bodies**	· Public knowledge of funded study (that is, use of funding).	· Widespread access to protocol.	· Confidence that protocol will not be plagiarized.	· Less certainty that study will be conducted as funded.
		· Protocol could be adapted or plagiarized by other investigators.	· Others (competitors/public) will not have easy access to funded study protocol.	
	· Greater accountability of investigators to adhere to funded protocol.			
	· Greater certainty that study will be conducted as funded.			
**Site collaborators**	· Availability of full protocol if they wish to seek that information.	· May believe that the protocol is open to change.	· Availability of full protocol if they wish to seek that information, but only if willing to sign a NDA that has been first agreed upon by their institution and the lead investigator’s institution.	· Cannot share details of the protocol with colleagues unless they are involved in the study.
	· Can share the protocol with other potential collaborators within their institution.			
				· Cost (time/effort) involved in review and approval of NDA, even when the site subsequently decides not to participate.
	· More open discussion.			
**Collaborators**’ **institutions** (**universities**, **health care organisations**)	· Availability of full protocol if they wish to seek that information.	· May believe that the protocol is open to change.	· Availability of full protocol for review before joining the study if NDA is signed.	· Cost involved in legal review and approval of NDA.
				· Co-investigators’ institutions need to sign NDAs before being able to establish the trial in their centre.
**End**-**users of evidence** (**clinicians**, **policy makers**)	· High degree of rigour associated with adhering to protocol and reporting.	· Potential for delay in publication because of greater rigour required.	· The study is unlikely to be duplicated.	· Possibility of selective and biased reporting.
				· Possibility of adopting flawed evidence.
	· Can check that findings are appropriately reported.			
**Patients** (**potential trial participants**)	· Availability of full protocol if they wish to seek that information.	· Misinterpretation of open access information by those without sufficient background knowledge to understand it.	· Potential trial participants can be more confident the protocol is unique.	· Patients would need to ask specifically for a copy of the protocol if they wished to obtain details of the study.
	· Can check that results are presented as intended.			
				· Possibility of selective and biased reporting.
				· Possibility of flawed evidence being adopted.
**Society generally**	· Potential for replication of research, leading to availability of studies for inclusion in meta-analyses and more rigorous evidence to support practice.	· Potential for research being duplicated unnecessarily (waste of scarce resources).	· Trial is unlikely to be replicated.	· Possibility of flawed evidence being adopted.
	· Protocols of publicly funded research will be openly available to the public.			

*Note that an NDA refers to protection of the protocol before a centre joins a trial, but this table assumes ongoing protection of the protocol during the conduct of the trial.

To summarise, the main advantages of the open access approach are that the protocol is freely available to all stakeholders (including potential and active participants), permitting them to discuss the protocol widely with colleagues (and family in the case of participants), assess the quality and rigour of the protocol, determine the feasibility of conducting the trial at their centre, and after trial completion, to evaluate the reported findings based on a full understanding of the protocol. One side effect of this openness is that both lead investigators and site collaborators can have a feeling of ownership of the protocol and the research. A potential societal advantage would be that replication of the research would lead to additional evidence being available to guide clinical practice. The main disadvantage of this approach is the potential for plagiarism; however if that occurred, it should be both reasonably easy to identify because of the open access to the original trial protocol, as well as ensure that appropriate sanctions are used to deal with plagiarism (for example those recommended by COPE [11] and Tri-Agency Framework: Responsible Conduct of Research [12]). A more insidious possibility would be that a rival group might adopt the protocol with minor changes, to avoid being accused of plagiarism.

The main advantages of the restricted access approach are for the lead institution and nominated principal investigator who protect their intellectual property associated with the trial. The main disadvantage is that there can be only one named institution and investigator, even if the trial was developed by a multidisciplinary and multi-institution research team. This approach implies that the protocol is owned by the lead institution, thus reducing the collegiality between the lead investigators and also between the lead investigators and site collaborators. Other disadvantages lie in defining who ‘needs to know’ about the study protocol during the site evaluation of the study, and how much information would constitute a breach of confidence: it is unclear whether, for example, presentation of the protocol at departmental rounds could constitute a breach of NDA. These definitions should be clear, because NDAs declare the threat of legal action if the protocol is shared unnecessarily, although it is not evident what legal remedies might be applied. As well, the use of NDAs involves costs to the lead site and both potential and actual collaborators, because the agreement must be reviewed and signed by each site’s lead investigator, lawyers and institutional representatives before the site investigator can even evaluate the merits of the protocol and the feasibility of study participation at that centre. This extra step, before the protocol can be discussed locally, will slow the process of site recruitment, and may even discourage sites from joining a trial, particularly when the subsequent process of study approvals is well known to be onerous [13].

## Discussion

Even if a specific research question is of interest to more than one research group, the complex and unique features that make up a multicentre investigator-initiated trial, including the study design as well as the infrastructure needed to run the trial, and the distinctive group or network of investigators and collaborators, ensure that an individual trial is unlikely to be replicated exactly, or the IP expropriated, with or without the protection of NDAs. Replication of trials is likely to occur only for research questions that are deemed important by a number of independent groups, and the resulting research findings will benefit society by providing further evidence to inform best clinical practice.

In academia where research questions usually occur as a result of clinical controversy, it is often the case that similar research questions occur spontaneously. This scenario was well demonstrated during the past decade by trials investigating the use of multiple courses of antenatal steroids in women at risk of preterm delivery, with outcomes of interest being safety and effectiveness in reducing perinatal morbidity. That research question was investigated by several large research groups, who produced a number of trial designs that were unique but sufficiently similar to enable the evidence from the trials to be synthesised to provide good evidence to inform clinical practice [14]. Further, the investigators continue to contribute to ongoing debates about the role and long-term outcomes of antenatal corticosteroids. The use of NDAs might decrease the potential for such international collegiality and limit the value of evidence that is produced from individual trials.

The two peer-reviewed CIHR-funded trials considered here represent the highest standard of Canadian review and trial conduct. They illustrate contrasting approaches to managing intellectual property associated with study design, which can be broadly described as open access (CHIPS) and restricted access (FACT) to the protocols.

Both trials are registered with ISRCTN, and therefore some details of the protocols are openly available from that register. Unfortunately, despite increasing standardisation and widespread use of registries [15-17], many still regard them as providing insufficient detail about a particular trial [16,18,19], inadequate reporting of protocol amendments [19,20], and lack of quality assurance of investigator-entered data [16]. As well, trial publications often show discrepancies from details available in registries [17,21]. Selective publication of trials and individual outcomes can lead to bias [18,21-24], with statistically significant results being favoured [21,22,24]. As a result of these concerns about trial registries, several commentators have recommended that trial registration should be supplemented by protocol publication [19,25-27]. Early publication of the protocol would be incompatible with a restricted access approach.

In the past, protocol publication was only possible on a study website, as represented by CHIPS [8]. Although peer review and publication is promoted by leading journals such as *The Lancet* (where the CHIPS protocol is published [9]) not all of the protocol details are available. With the advent of open access journals, prospective publication of full study protocols in the public domain is becoming more common (for example in journals such as *Trials*[28] and Biomed Central publications [29]). Publication of protocols in open access journals is the most accountable way of ensuring that full details of a study are known. If protocols are made widely available, authors can be held to the highest standard of reporting [19,25], including details of changes to primary outcome or sample size. Even if protocols are published, final publications may deviate from the initial protocols [25-27,30], including peer-reviewed government-funded trials [22], and those published in high-quality journals [18]. Open access to protocols can help to ensure that discrepancies between original protocol and eventual publication are easily identified, even though bias cannot be entirely eliminated.

One constraint associated with publishing a protocol is that the investigators must be rigorous in making amendments to that initial protocol when there are legitimate reasons for making changes. Such changes can be addressed with an addendum to the protocol, and will be reported in the final publication. The point is that the details are transparent to journal editors and readers.

NDAs between the lead institution and sub-sites may represent a legally responsible approach to intellectual property protection. Unfortunately there are potential disadvantages: adding an extra legal step into sub-site recruitment will make this process more difficult; NDAs may reduce academic openness and collegiality between potential and actual investigators; and restricting the availability of the protocol could allow investigators to present selected results. In the case of the FACT Trial, the use of NDA was short-lived. The additional hurdle of the NDA was widely criticized by potential collaborators, and the principal investigators (including MW) insisted that the lead institution stop using this approach.

The use of NDAs is an increasing trend in Canada. If this trend continues, it will have an impact on the work of clinical trialists, perhaps making even more difficult site recruitment and progress to full site activation. It is well documented that poor recruitment to trials is the main reason trials fail [31,32], and it is possible that the introduction of NDAs may contribute to the failure of well-designed trials without any significant benefit in protecting intellectual property. In institutions where legal departments require the use of NDAs, investigators should be prepared to negotiate with their institution to remove this requirement if they believe that the use of NDAs will harm their trial. Public funding agencies should formalize rules around open versus restricted access to study protocols of publicly funded trials, just as they have with regards to open access to results [33-35].

## Competing interests

SR is a member of the steering committee of the CHIPS Study. LM is principal investigator of the CHIPS Study. MW is principal investigator of the FACT Study. SW is Calgary site investigator for the FACT Study.

## Authors’ contributions

SR, LM and SW played a major role in the conception of the commentary. SR, LM, MW and SW contributed to the writing, and have read and given final approval of the manuscript as submitted. All authors read and approved the final manuscript.
